# Skin‐Protective Performance of Alternative Stratum Corneum Formed by a Pseudo‐Ceramide‐Containing Steroid Lamellar Cream

**DOI:** 10.1111/exd.70041

**Published:** 2025-03-11

**Authors:** Masafumi Yokota, Tomohiro Matsumoto, Akane Kawamoto, Kumiko Dojo, Sumika Toyama, Catharina Sagita Moniaga, Junko Ishikawa, Daiki Murase, Noriyasu Ota, Mitsutoshi Tominaga, Kenji Takamori

**Affiliations:** ^1^ Juntendo Itch Research Center (JIRC), Institute for Environmental and Gender‐Specific Medicine Juntendo University Graduate School of Medicine Chiba Japan; ^2^ Biological Science Research Kao Corporation Kanagawa Japan; ^3^ Biological Science Research Kao Corporation Tochigi Japan; ^4^ Department of Dermatology Juntendo University Urayasu Hospital Chiba Japan

**Keywords:** allergen, ceramide, proteases, pruritus, skin barrier function

## Abstract

Ceramides in the stratum corneum (SC) are important for epidermal barrier function. We previously developed a synthetic pseudo‐ceramide for medical (SPCM)‐containing steroid cream [SPCM (+)]. This cream forms films on the skin surface and exerts anti‐inflammatory effects through steroids. However, the preventive effects of this cream on the disruption of the skin barrier remained unclear. Therefore, in this study, we aimed to evaluate the protective role of SPCM (+) cream against atopic dermatitis (AD)‐associated protease allergens on the skin in recovery from barrier‐broken skin. We used three‐dimensional (3D) skin and mouse models of disrupted skin barriers to evaluate the protective effect of SPCM (+) cream against V8 protease produced by *Staphylococcus aureus* . In NC/Nga mice with itching caused by living mites, SPCM (+) cream was repeatedly applied once a day for 2 weeks, and scratching behaviour was assessed every week using the MicroAct system. In the 3D skin model, the SPCM (+) cream directly blocked SC degradation by V8 protease of *S. aureus* and suppressed the expression of interleukin‐36 gamma. The application of SPCM (+) cream to mite‐parasitised mice suppressed scratching, reduced elevated activity of skin proteases, and inhibited upregulation of thymic stromal lymphopoietin. These beneficial effects of SPCM (+) cream were not observed with steroid creams without SPCM. These results suggest that the SPCM (+) cream is effective in relieving inflammation and itching by protecting the skin from proteases and allergens through its lamellar structure. This cream may be a promising treatment option for skin barrier disorders including AD and xerosis.

## Graphical abstract

The lamellar structure‐forming cream with a steroid and medical pseudo‐ceramide functioned as a substitute for the stratum corneum and showed the improvement of skin barrier function and anti‐inflammatory and antipruritic effects. This cream may be a promising therapeutic option for barrier‐disrupted skin diseases, such as atopic dermatitis and xerosis.

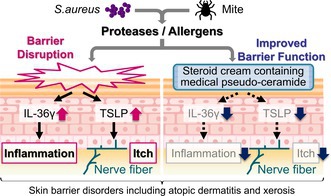

## 1 Introduction

The epidermis protects the body from environmental stress triggered by physical, chemical, and pathological factors and acts as a barrier to prevent water loss from the body. Intercellular lipids, known as ceramides, play an essential role in the skin barrier function. Ceramides contribute to the integrity of the epidermal barrier by organising lipid multilayers (lamellar) together with cholesterol and free fatty acids [1]. Shortage and aberrant profiles of ceramides in the stratum corneum (SC) are closely linked with the pathogenesis of atopic dermatitis (AD) and psoriasis, which are mainly characterised by impairment of the skin barrier [2, 3].

Improving skin barrier function is considered very important in AD because penetration by external irritants causes the onset or relapse of inflammation [4]. Harmful components commonly associated with AD exacerbation are abundant in the environment, and allergens derived from *Staphylococcus aureus* , house dust mites, and pollen are well known [5]. Most allergens possess proteolytic activity (protease allergens) and can disrupt the epidermal barrier. Moreover, protease allergens and mechanical barrier damage, such as that caused by scratching, collaboratively facilitate epicutaneous sensitization, skin inflammation, such as AD, and atopic march including asthma [6]. Consequently, ameliorating impaired skin barrier function and protecting skin from allergens are important for skin and systemic health.

The topical application of ceramides to skin lacking intercellular lipids and with an impaired skin barrier restores barrier function and the water content of the SC [7, 8]. However, extraction from living organisms or the chemical synthesis of natural ceramides for skin application poses serious safety and cost related challenges [9]. Natural ceramides derived from biogenic sources are primarily extracted from bovine pituitary (bovine pituitary extracts, BPE), and prion‐contaminated BPE can transmit bovine spongiform encephalopathy (BSE) [10]. Although synthetic ceramides do not carry the risk of infectious diseases like BSE, producing skin‐equivalent ceramides is highly expensive (2000–several hundred thousand €/kg) [11]. The commercial use of natural ceramides derived from plants and yeast has been growing in recent years; however, issues regarding production, such as low yields and difficulties in extraction and purification, need to be resolved [12, 13].

Therefore, pseudo‐ceramides mimicking skin‐identical ceramides, such as PC‐104 {N,N′‐(2‐hydroxy‐1,3‐propanediyl)bis[N‐(2‐hydroxyethyl)hexadecanamide]} and Bio391 [N‐(2‐hydroxyethyl)‐2‐pentadecanolyl hexadecanamide], have been developed and tested. These pseudo‐ceramides are relatively cheap to produce and less‐toxic; in addition, creams and lotions containing pseudo‐ceramides effectively improve skin barrier function in cases of xerosis and AD, as well as those containing skin‐derived ceramides [9, 14, 15]. SPCM [N‐(3‐hexadecyloxy‐2‐hydroxypropyl)‐N‐2‐hydroxyethyl hexadecanamide] is one of the synthetic pseudo‐ceramides, and mimics naturally occurring ceramide type 2 (NS) [16]. Supplementation with SPCM restores barrier integrity and water‐holding properties of the SC, thereby exhibiting therapeutic efficacy against experimental barrier‐disrupted and dry skins of patients with AD [16, 17]. Recently, we have developed an oil‐in‐water cream by combining a steroid (prednisolone valerate acetate, PVA) with SPCM. This SPCM‐containing steroid cream [SPCM (+) cream] has improved disease severity to the same extent as that of commercial steroid creams after 2 weeks of application in patients with AD. Notably, SPCM (+) cream substantially restores skin properties, such as moisture content of the SC and transepidermal water loss (TEWL), more quickly than the steroid cream lacking SPCM [18]. Formulations with multilamellar structures behave like SC with appropriate moisture permeability while limiting substance penetration [19, 20]. These findings imply that the protective effect of the lamellar structure may play an important role in the efficacy of SPCM (+) cream and in replenishing SPCM and anti‐inflammatory effects of steroids. However, the mechanism by which the alternative SC formed by SPCM (+) cream contributes to skin restoration is not well understood.

In the present study, we focused on the harmful effects of AD‐associated protease allergens on skin and evaluated the potential of SPCM (+) cream to recover barrier‐broken skin, similar to that of AD, using in vitro and in vivo models. Herein, we describe how the SPCM (+) cream suppresses the expression of cytokines, such as interleukin‐36 gamma (IL‐36γ) and thymic stromal lymphopoietin (TSLP), by protecting skin from proteases, as an alternative SC, and alleviates skin inflammation and itching.

## 2 Methods

### 2.1 Test Creams

In this study, synthetic pseudo‐ceramide of medical‐use grade was used. A synthetic pseudo‐ceramide for medical (SPCM) was provided by Kao Corporation (Tokyo, Japan). The chemical structure of SPCM is shown in Figure S1. The SPCM (+) cream was an oil‐in‐water formulation containing 0.15% prednisolone valerate acetate (PVA) and 3% SPCM. The oil‐in‐water formulation was prepared by mixing the aqueous and oil phases at 80°C–85°C and cooling to room temperature. Other components included cetyl alcohol, stearyl alcohol, polyoxyethylene sorbitan monostearate, sorbitan monostearate, glycerin, and water. The control cream [SPCM (−) cream] was formulated by replacing SPCM with water. The cream containing no SPCM or PVA was designated as the vehicle cream (Vehicle).

### 2.2 Reagents

Mouse anti‐CDSN antibody was purchased from Abcam (Cambridge, UK). Alexa Fluor 488‐conjugated donkey anti‐mouse IgG antibody, Dulbecco's Modified Eagle Medium (DMEM) high glucose, and Ham's F‐12 Nutrient Mix were purchased from Thermo Fisher Scientific (Waltham, MA, USA). *S. aureus* V8 protease was purchased from FUJIFILM Wako Pure Chemical (Osaka, Japan) and dissolved in Dulbecco's phosphate‐buffered saline (PBS, Thermo Fisher Scientific) for use.

### 2.3 Structural Observation by Transmission Electron Microscopy

The test cream was applied onto a polyethylene terephthalate film using a coater (thickness, 120 μm). After 3 h of drying, they were cut into 5‐mm squares, stained, and fixed with osmium tetroxide for 24 h at 23°C. A Leica UC‐6 cryo‐ultramicrotome (Leica Microsystems, Wetzlar, Germany) was used to obtain ultrathin sections of 50–100 nm thickness at −80°C. The sections were collected in droplets of frozen 2.3 M sucrose, placed on a grid (200 mesh with film without carbon reinforcement), washed and dried. Transmission electron microscopy was performed using an H‐7650 (Hitachi, Tokyo, Japan) at an accelerating voltage of 80–100 kV. A charge‐coupled device (1024 × 1024 pixels) was used for detection.

### 2.4 Cell Culture

A three‐dimensional (3D) human epidermal model (LabCyte EPI‐MODEL12 6D) was obtained from Japan Tissue Engineering Co. Ltd. (Aichi, Japan). Cells were pre‐incubated overnight with culture media provided by the manufacturer at 37°C in a humidified atmosphere of 95% air and 5% CO_2_. After preincubation, the medium was replaced with conditioned medium consisting of a 3:1 mixture of DMEM and Ham's F‐12 medium. Each cream was applied topically to the 3D skin model and dried for 30 min. Subsequently, filter paper absorbed with 60 μL PBS or 1% (*w*/*v*) V8 protease solution was overlaid on the coated cream, and cells were harvested after 3 h.

### 2.5 Immunostaining

Frozen blocks were prepared by embedding unfixed skin tissues in Super Cryoembedding Medium (Leica Microsystems, Wetzlar, Germany). Next, 6‐μm‐thick cryosections were cut using a Cryostat Microm HM550 (Thermo Fisher Scientific), dried, and fixed in 4% paraformaldehyde for 4 h. The sections were blocked with Protein Block Serum‐Free Ready‐to‐use (Agilent Technologies, Santa Clara, CA, USA) for 10 min at 25°C and then incubated with primary antibody at 4°C overnight. The secondary antibody and 4′,6‐diamidino‐2‐phenylindole (DAPI) solution (Thermo Fisher Scientific) were added to the sections and incubated at 25°C for 30 min, followed by the addition of Fluoromount‐G (Southern Biotech, Birmingham, AL, USA). Antibodies and DAPI were diluted using Can Get Signal Solution A (TOYOBO, Osaka, Japan). Immunofluorescent images were captured using a confocal fluorescence microscope LSM710 and imaging software ZEN v.3.1 (blue edition) (Carl Zeiss Microscopy, Jena, Germany).

### 2.6 mRNA Extraction and Quantitative Real‐Time Polymerase Chain Reaction (PCR)

Total RNA was extracted from cultured cells and mouse tissues using an RNeasy Mini Kit (QIAGEN, Venlo, the Netherlands) and reverse‐transcribed using a High‐Capacity RNA‐to‐cDNA kit (Thermo Fisher Scientific). Quantitative real‐time PCR was performed on a 7500 Fast Real‐Time PCR System (Thermo Fisher Scientific) using TaqMan Fast Universal PCR Master Mix (Thermo Fisher Scientific). The following human and mouse genes were analysed using TaqMan Gene Expression Assays (Thermo Fisher Scientific): human *RPLP0* (Hs00420895_gH), mouse *Rplp0* (Mm00725448_s1), human *IL36G* (Hs00219742_m1), human *CXCL8* (Hs00174103_m1), human *MMP9* (Hs00957562_m1), human *DEFB4B* (Hs00823638_m1), and mouse *Il36g* (Mm00463327_m1).

### 2.7 RNA Sequencing (RNA‐Seq)

Sequencing libraries were prepared using an Ion AmpliSeq Transcriptome Human Gene Expression Kit (Thermo Fisher Scientific). Briefly, RNA was reverse‐transcribed using a SuperScript VILO cDNA Synthesis Kit (Thermo Fisher Scientific). cDNA was ligated with Ion Xpress Barcode adaptors and purified using AMPure XP beads (Beckman Coulter, Brea, CA, USA). A quality check of the library was performed using a High Sensitivity D1000 ScreenTape on an Agilent 4200 TapeStation. Each library was quantified using an Ion Library TaqMan Quantitation Kit (Thermo Fisher Scientific) and diluted to 100 pM prior to template preparation. Templates were prepared and loaded onto an Ion 540 chip in an Ion Chef System (Thermo Fisher Scientific). RNA‐seq was performed using an Ion S5 XL System (Thermo Fisher Scientific).

### 2.8 RNA‐Seq Data Analysis

Read count data were generated using the AmpliSeq RNA plug‐in of the Ion Torrent Suite Software v.5.14.0 (Thermo Fisher Scientific). Differentially expressed genes (DEGs) were analysed using integrated Differential Expression and Pathway analysis (iDEP) v.0.94. iDEP is a web‐based tool for analysing RNA‐seq data based on the R/Bioconductor packages [21]. DEGs were defined with a false discovery rate < 0.05, and fold‐change > 1.5.

### 2.9 Enzyme‐Linked Immunosorbent Assay

Whole mouse skin was homogenised and sonicated using a CelLytic MT Cell Lysis Reagent (Sigma‐Aldrich, St. Louis, MO, USA) supplemented with a protease/phosphatase inhibitor cocktail (Cell Signaling Technology, Danvers, MA, USA). After centrifugation (15 000 × *g* for 15 min at 4°C), the supernatants were collected. Concentrations of IL‐36γ and TSLP were determined according to the manufacturer's instructions. Protein quantity was determined using a BCA protein assay kit (Thermo Fisher Scientific). The amounts of cytokines were adjusted according to protein quantity of the supernatants.

### 2.10 Mice

Male ICR mice (eight‐week‐old) and male NC/Nga mice (eight–nine‐week‐old) were purchased from Japan SLC (Shizuoka, Japan). These mice were kept under controlled conditions with a 12‐h light/12‐h dark cycle and a steady temperature of 23°C ± 2°C and had ad libitum access to water and food. All animal experiments were approved by the Animal Ethics Committee of Kao Tochigi Institute (approval number: F17079‐0001, K20037‐01), Juntendo University (approval number: 2022112), and Japan SLC (approval number: E77‐8111).

### 2.11 Skin Barrier Disruption and Protease‐Challenge Model

Three days after shaving the rostral backs of mice, cotton soaked in a mixture of acetone and diethyl ether (1:1) was applied to the shaved backs for 15 s, immediately followed by soaking with distilled water for 30 s [acetone‐ether‐water (AEW) treatment]. The treatment was repeated twice daily at intervals of more than 6 h for 2 days. AEW treatment was followed by cream application and exposure to V8 protease. 40 μL of each cream were applied to the shaved area (2 × 2 cm) and dried for 1 h. Using a syringe equipped with a nasal nebulizer (Fuji‐Medical Corporation, Tokyo, Japan), 0.02% (*w*/*v*) V8 protease solution was sprayed onto the cream‐treated area and dried for at least 30 min. These processes were continued for 3 days. All treatments were performed under isoflurane anaesthesia (Viatris Inc., Canonsburg, PA, USA).

### 2.12 Measurement of TEWL

TEWL was evaluated using a Tewameter TM300 (Courage and Khazawa, Cologne, Germany). Measurements were conducted by placing the device on the surface of the back skin under isoflurane anaesthesia.

### 2.13 Mite‐Infested AD Model

NC/Nga mice develop AD‐like dermatitis under conventional circumstances, but not under specific pathogen‐free (SPF) conditions [22]. In this study, dermatitis was induced in mice parasitised with *Myobia musculi* in a conventional environment to increase the efficiency of this model [23] and to evaluate the protective effect of each cream against mite proteases. Each cream (50 mg) was topically applied to the shaved back (2 × 2 cm) and ears once daily for 2 weeks. Mice were anaesthetised with isoflurane (Viatris Inc.) during the experimental procedure.

### 2.14 Quantification of Scratching Behaviours

Scratching behaviour was automatically and objectively recorded using MicroAct (Neuroscience, Tokyo, Japan) as previously described [24]. Briefly, a Teflon‐coated magnet (1.0 mm in diameter, 3.0 mm in length) was subcutaneously implanted into the hind paws of isoflurane‐anaesthetised mice 3 days before measurement. Each mouse was placed in an observation chamber surrounded by a circular coil containing food and water. Scratching behaviour was defined as electric current in the coils induced by the movement of magnets in the hind paws. The analysis parameters for detecting scratch motions are listed in Table S1. The number of scratching events was counted over 22 h.

### 2.15 Skin Protease Activity Assay

Protease activity in the skin was assessed using an EnzCheck Protease Assay Kit (Thermo Fisher Scientific) consisting of casein derivatised with a fluorescent dye (casein‐FL). Although undigested casein‐FL is quenched, casein‐FL cleaved by proteases can fluoresce. The lesion skin of NC/Nga mice was dissected, homogenised, and sonicated in a buffer supplied by the manufacturer. The supernatant was separated by centrifugation (15 000 × *g* for 15 min at 4°C) and used for the assay. Protease activity was measured immediately after mixing the supernatant with casein‐FL over a period of 60 min. Fluorescence was measured at 485 nm (excitation) and 530 nm (emission) using a plate reader SH‐9000 (Hitachi High‐Tech Corporation, Tokyo, Japan).

### 2.16 Statistical Analysis

Data are presented as the mean ± standard error (SEM). Student's *t*‐test and one‐way analysis of variance with Dunnett's or Tukey's multiple comparison tests were used for statistical analyses. All analyses were performed using GraphPad Prism v.8 (GraphPad Software, La Jolla, CA, USA).

## 3 Results

### 3.1 Lamellar Structure Formed by the SPCM (+) Cream Mitigated Damage to the Epidermal Barrier by V8 Protease

**FIGURE 1:**
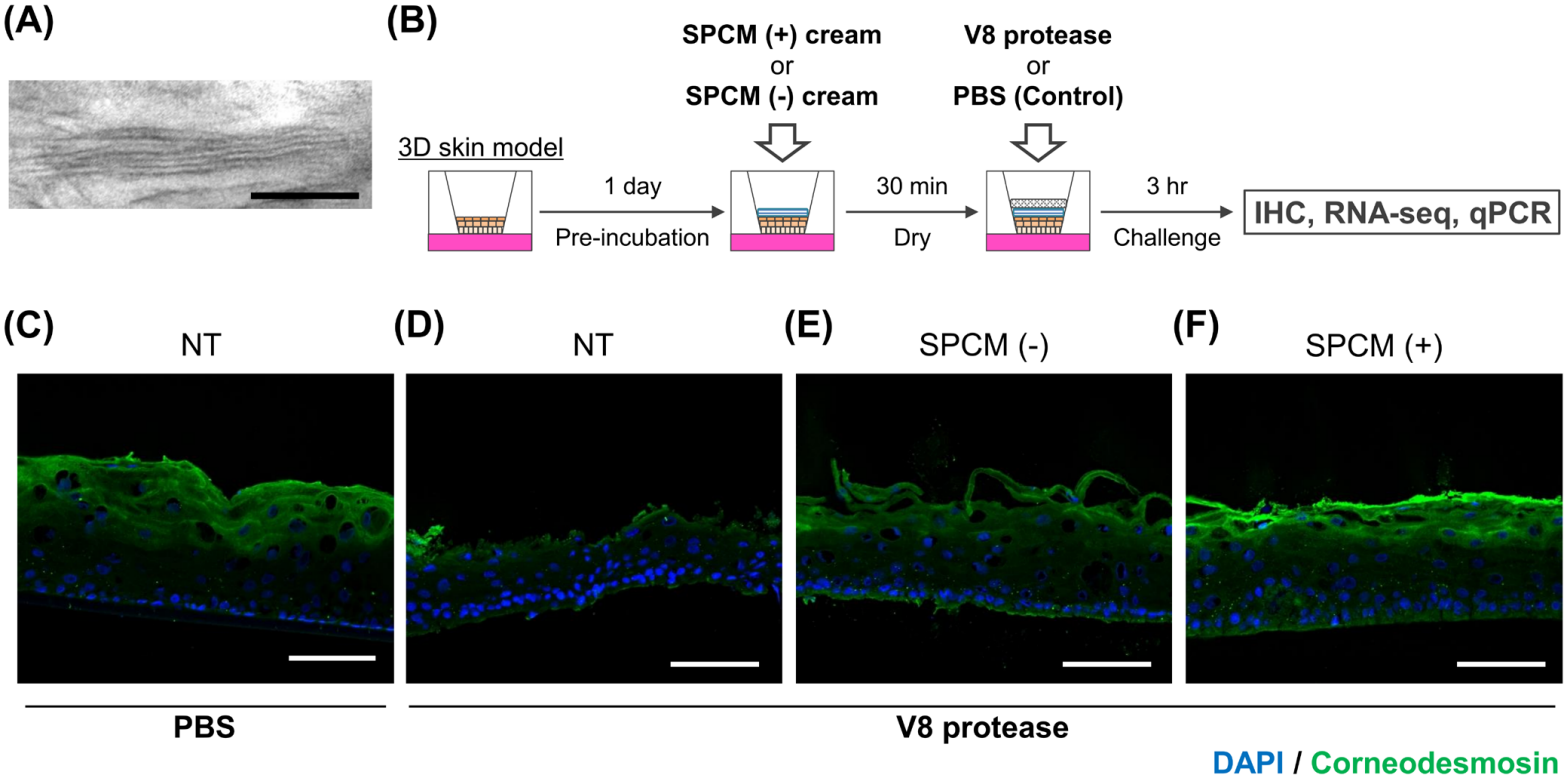
Lamellar structure of SPCM‐containing cream and its protective effect against V8 protease. (A) Representative transmission electron microscopic images of lamellar structure formed by the SPCM (+) cream. Scale bar, 100 nm. (B) Scheme of the experiment using a reconstructed human epidermis model. (C–F) Representative fluorescence images of 3D skin models stained with anti‐CDSN antibody (SC marker, green) and DAPI (nuclear marker, blue). Scale bars, 100 μm. CDSN, corneodesmosin; NT, untreated samples; PVA, prednisolone valerate acetate; SC, stratum corneum; SPCM (−), PVA cream without SPCM; SPCM (+), SPCM‐containing PVA cream; SPCM, synthetic pseudo‐ceramide for medical.

Lamellar structure formed by the SPCM (+) cream was confirmed using transmission electron microscopy (Figure 1A). However, no lamellar structure was observed for the SPCM (−) cream (data not shown). We conducted experiments using a 3D skin model (Figure 1B) to evaluate the protective effect of SPCM (+) cream against V8 protease produced by *S. aureus* [25]. V8 protease is involved in the pathogenesis of AD by causing disruption of the skin barrier and inflammation [26, 27]. The influence of V8 protease on the SC was evaluated by immunostaining based on the fluorescence intensity of corneodesmosin (CDSN) (Figure 1C). Exposure of the 3D skin model to V8 protease without any treatment resulted in the disappearance of the SC (Figure 1D). In the SPCM (−) cream‐treated group, the CDSN signal was attenuated, and the surface of the SC was partially stripped, indicating that the SC structure was considerably damaged (Figure 1E). No damage to the SC was observed in the SPCM (+) cream‐treated group, which was comparable to that observed in the V8 protease‐untreated group (Figure 1F).

### 3.2 IL‐36γ, an Inflammatory Cytokine, Reflected Skin Response to V8 Protease

**FIGURE 2:**
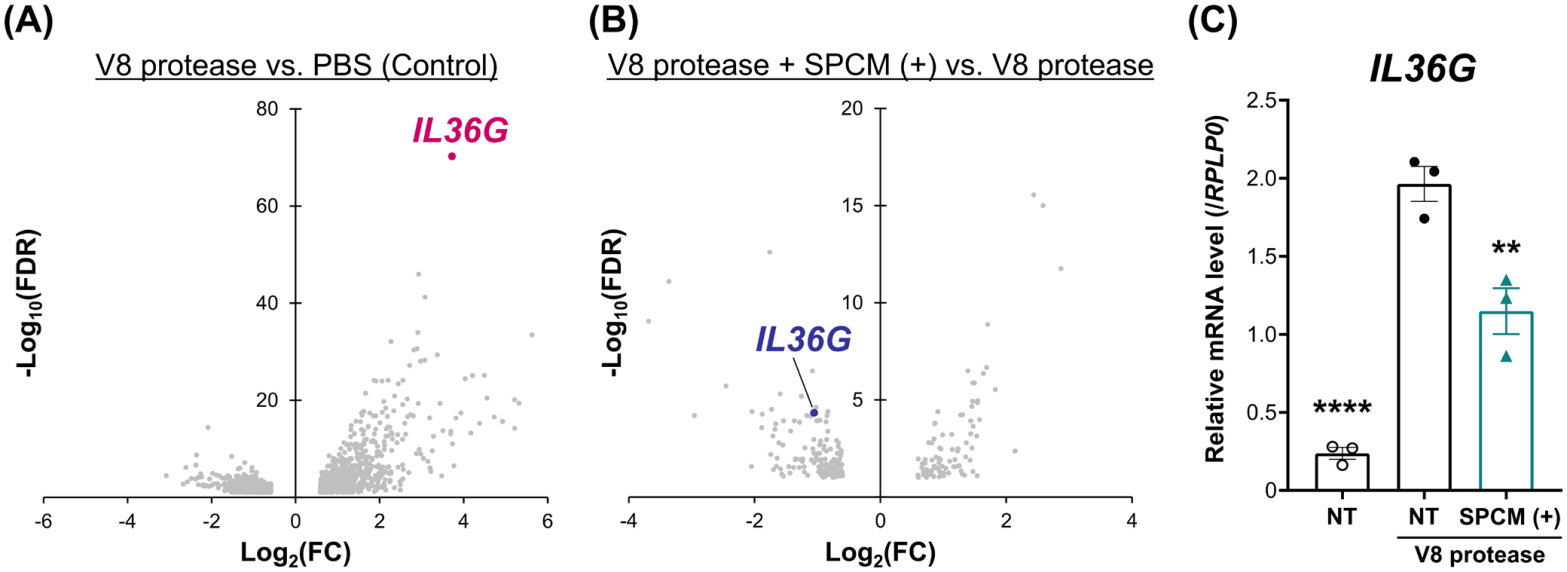
IL‐36γ is a marker of V8 protease‐induced SC. (A, B) Volcano plot of DEGs in the V8 protease‐treated group vs. control group (A) and V8 protease + SPCM (+)‐treated group vs. V8 protease‐treated group (B). Upregulated genes are towards the right, and downregulated genes are towards the left [*n* = 3, Benjamini–Hochberg false discovery rate (FDR) < 0.05, fold change (FC) > 1.5]. (C) *Il36g* mRNA expression was validated by quantitative real‐time PCR. *n* = 3. Data represent mean ± SEM. ****p < 0.0001, **p < 0.01, Dunnett's test (vs. NT under V8 protease challenge). IL‐36γ, interleukin‐36γ; NT, untreated samples; SC, stratum corneum; SPCM (+); SPCM‐containing PVA cream; SPCM, synthetic pseudo‐ceramide for medical.

Next, we analysed gene expression in 3D skin models using RNA‐seq. DEGs analyses between PBS‐ and V8 protease‐treated samples revealed that *IL36G* was markedly upregulated by V8 protease (Figure 2A). Analysis of DEGs between groups with and without SPCM (+) cream treatment under V8 protease stimulation showed that *IL36G* expression was markedly decreased in the SPCM (+) cream‐treated group (Figure 2B). We further validated the RNA‐seq data using real‐time quantitative PCR and obtained data consistent with that of the RNA‐seq analysis (Figure 2C). In addition to *IL36G*, C‐X‐C motif chemokine ligand 8 (*CXCL8*), matrix metalloproteinase 9 (*MMP9*), and defensin beta 4B (*DEFB4B*) were identified as candidate markers for skin response to V8 protease (Figure S2). However, these are downstream genes of the IL‐36γ signalling pathway [28, 29] and *CXCL8* is deleted in the mouse and rat genomes [30], limiting further investigation. For these reasons, we selected the IL‐36γ expression as a suitable marker for evaluating skin reactions to V8 protease.

### 3.3 The SPCM (+) Cream Conferred Barrier Function on the Impaired Skin

**FIGURE 3:**
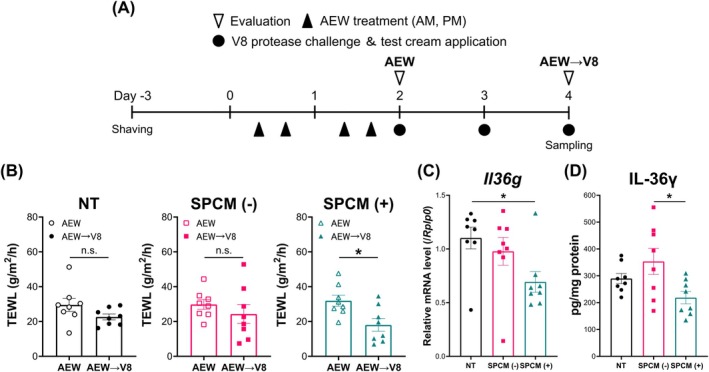
The SPCM‐containing cream serves as a substitute of SC on the disrupted skin. (A) Scheme of the experiment of skin barrier disruption and protease challenge model. (B) Changes in skin barrier function (TEWL) of the SC before and after application of the test cream. *n* = 8. Data represent mean ± SEM. *p < 0.05, Paired *t*‐test. (C, D) The mRNA (C) and protein (D) expression of IL‐36γ after V8 protease stimulation along with treatment of the test cream. *n* = 8. Data represent mean ± SEM. *p < 0.05, Tukey's test. IL‐36γ, interleukin‐36γ; n.s., not significant; NT, untreated samples; SC, stratum corneum; SPCM (−), PVA cream without SPCM; SPCM (+), SPCM‐containing PVA cream; SPCM, synthetic pseudo‐ceramide for medical; TEWL, transepidermal water loss.

Both AEW treatment and the application of V8 protease break the epidermal barrier in mice [31, 32]. Therefore, we designed an experimental model that combined AEW treatment and V8 protease exposure to investigate the efficacy of SPCM (+) cream on barrier‐impaired skin (Figure 3A). TEWL was significantly reduced in the SPCM (+) cream‐treated group compared to that in groups after AEW treatment (Day 2) and V8 protease application (day 4) (*p* = 0.0483) (Figure 3B). IL‐36γ levels in the V8 protease‐sprayed and unsprayed areas reflected the effect of V8 protease on murine skin, which was similar to that of the 3D skin model (Figure S3). To determine whether protection against V8 protease was associated with the recovery of TEWL, we compared the IL‐36γ expression among groups. IL‐36γ expression was suppressed in the SPCM (+) cream‐treated group compared to that in the untreated and SPCM (−) cream‐treated groups (Figure 3C,D).

### 3.4 Regular Use of the SPCM (+) Cream Suppressed Scratching Behaviour in AD Mice

**FIGURE 4:**
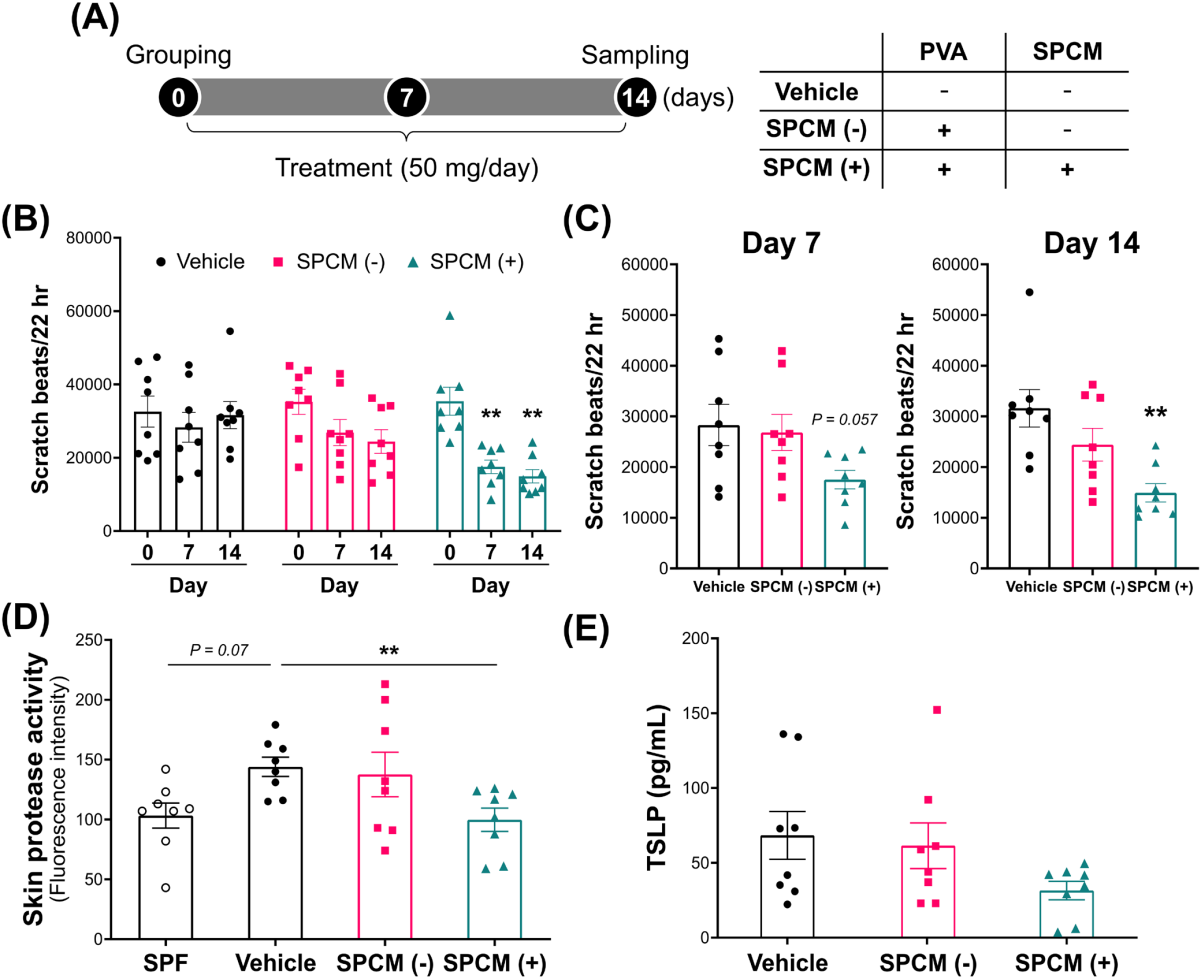
Antipruritic effect of the SPCM‐containing cream in AD model mice. (A) Scheme of the experiment using mite‐infested AD model mice and overview of test cream. (B) Changes of scratch bouts during 2 weeks of treatment with the test cream. *n* = 8. Data represent mean ± SEM. **p < 0.01, Dunnett's test (vs. day 0). (C) Comparison of scratch bouts on days 7 and 14. *n* = 8. Data represent mean ± SEM. **p < 0.01, Dunnett's test (vs. Vehicle). (D) Protease activity in the skin of AD model mice. SPF mice were not infested by mite, and no AD manifestations were observed. *n* = 8. Data represent mean ± SEM. **p < 0.01, Dunnett's test (vs. Vehicle). (E) TSLP production in the skin of AD model mice. TSLP is an itch mediator mainly secreted by keratinocytes. AD, atopic dermatitis; SPCM (−), PVA cream without SPCM; SPCM (+), SPCM‐containing PVA cream; SPCM, synthetic pseudo‐ceramide for medical; TSLP, thymic stromal lymphopoietin; Vehicle, control cream with neither SPCM nor PVA.

Similar to V8 protease, mite proteases adversely affect skin [5]. As allergens, they also contribute to the development of AD, and their relationship with pruritus has been reported [33]. Therefore, we hypothesised that the SPCM (+) cream would prevent itching triggered by mite‐derived substances. Using NC/Nga mice with itching caused by living mites, scratching behaviour was assessed every week, and each cream was repeatedly applied once a day for 2 weeks (Figure 4A). The frequency of scratching bouts did not change in the Vehicle‐treated group, whereas scratching behaviours significantly decreased in the SPCM (+) cream‐treated group from the first week onwards. Treatment with the SPCM (−) cream also reduced scratching behaviour, albeit to a lesser extent than that by the SPCM (+) cream‐treated group (Figure 4B). Comparison among the groups at each time point showed that the application of SPCM (+) cream only suppressed itching in the first week, and this trend was more evident in the second week (Figure 4C).

### 3.5 Protease Activity and TSLP Expression in the Skin of AD Mice Were Reduced by Treatment With the SPCM (+) Cream

Mite‐derived proteases evoke itching by activating protease‐activated receptor 2 (PAR2) [34]. Therefore, we focused on skin protease activity to determine the mechanism underlying the inhibition of itching by the SPCM (+) cream. Skin protease activity increased in the Vehicle‐treated mice relative to that in the SPF mice. The SPCM (+) cream‐treated group displayed a significant reduction in skin protease activity (Figure 4D). TSLP is a representative itch mediator secreted by keratinocytes, and its release is attributed to PAR2 activation in keratinocytes [35]. The SPCM (+) cream‐treated group exhibited the lowest TSLP level among all groups (Figure 4E).

## 4 Discussion

In the present study, using 3D skin models, dry skin and AD model mice, we found that the topical application of the SPCM (+) cream to the skin with weakened barrier function suppressed inflammation (increased levels of IL‐36γ and TSLP), disruption of skin barrier function (increase in TEWL), and itching (increased number of scratching bouts), by preventing the entry of foreign proteases and living mites into the body. Because we observed that the SPCM (−) cream treatment partially suppressed the degradation of SC by V8 protease, the SPCM (−) cream can be expected to have a certain protective effect. However, the CDSN signal in the SPCM (−) cream‐treated group was obviously attenuated and a part of the SC detached compared to observations in the SPCM (+) cream‐treated group. These findings indicate that mixing the cream with SPCM provided protective effects other than the ability to cover the skin surface. The dried film of SPCM (+) cream formed regular layers at approximately 10‐nm intervals. Periodic lamellar organisation of intercellular lipids of the human SC with an interval of 6–13 nm were very similar with the structure formed by the SPCM (+) cream [36]. The lamellar phase present in intercellular lipids plays an important role in maintaining the barrier function and moisture content of the SC; therefore, SPCM (+) cream presumably functioned as a substitute for the SC on the skin. However, because the components of SPCM (+) cream and actual SC are quite different, more in‐depth studies are required to determine whether the lamellar structure in SPCM (+) cream plays a predominant role in its protective effect as in the natural SC. These findings suggest that the SPCM (+) film shields the skin from extrinsic proteases and prevents inflammation and itching in a wide range of skin conditions, from healthy to damaged skin.

RNA‐seq analysis of the 3D skin model revealed that IL‐36γ was produced in response to V8 protease stimulation. IL‐36γ is a proinflammatory cytokine expressed in the epidermal, bronchial, and intestinal epithelial layers [29, 37]. IL‐36γ is upregulated in various inflammatory skin diseases, such as psoriasis, and is considered to be a major driver of skin inflammation [38]. Other V8 protease‐responsive genes (*CXCL8*, *MMP9* and *DEFB4B*) in this study were all upregulated upon IL‐36γ stimulation of the epidermis [28, 29], suggesting that IL‐36γ also plays a critical role in the biological response of skin to V8 protease. These findings highlight the clinical importance of the SPCM (+) cream in inhibiting IL‐36γ production in V8 protease‐treated skin. In addition to IL‐36γ, interleukin‐1β and human β‐defensin 2 have been detected as V8 protease‐responsive molecules in the human keratinocyte cell line HaCaT [39]. These results suggest that V8 protease disrupts the SC and affects the epidermal layer. *S. aureus* secretes various proteases, including V8 protease, to degrade the epidermal barrier and penetrate the human skin [26]. *S. aureus* is frequently detected deep inside the SC of patients with AD, and the amount of *S. aureus* in the SC is correlated with disease severity [27]. As physical barriers such as multilamellar layers of the SPCM (+) cream are expected to be effective against *S. aureus* proteases, the SPCM (+) cream may be useful for patients with AD under skin conditions where *S. aureus* is likely to colonise the deep cutaneous layers, to maintain skin health.

In the skin barrier disruption and protease challenge models, the SPCM (+) cream significantly reduced TEWL, whereas the SPCM (−) cream did not affect TEWL. A moist environment facilitates epidermal turnover, thereby quickly normalising the function of SC. The film formed by the SPCM (+) cream exhibits high moisture retention and occlusive properties, and these effects are at least partly attributed to its lamellar structure on the skin [20]. AEW treatment transiently elicits skin dryness, and the water‐retention capability of the SPCM (+) cream is expected to restore the epidermal barrier. Compared to that of the SPCM (−) cream‐treated group, IL‐36γ expression in the skin was downregulated in the SPCM (+) cream‐treated group. Aberrantly enhanced IL‐36γ signalling exacerbates tissue damage, and downregulation of IL‐36γ function promotes wound repair [40, 41]. Therefore, suppression of IL‐36γ expression by the SPCM (+) cream may partly contribute to improving the skin barrier. An SPCM lamellar cream designed with the same strategy was reported to positively change the ceramide profile of the SC and improve TEWL [42]. According to this study, the penetration of SPCM from the SPCM (+) cream into the damaged SC may also have contributed in part to the skin barrier restoration. However, the primary mechanism of skin barrier recovery is unknown, thus necessitating further detailed research, such as measurement of SPCM penetration into the SC.

Behavioural analyses showed that treatment with the SPCM (+) cream significantly inhibited scratching behaviour in mite‐infested AD model mice. Skin disorders with chronic itching, such as AD, psoriasis, and xerosis, lead to a vicious itch‐scratch cycle, in which frequent scratching aggravates skin symptoms and further intensifies itching [43]. To overcome this negative cycle, one rational therapeutic strategy to reinforce skin integrity is to reduce scratching. Although PVA included in the SPCM (+) and SPCM (−) creams is a relatively weak steroid, its usefulness in relieving pruritus in AD is well established [44]. From permeability tests using porcine skin, we verified that transdermal permeability of PVA was comparable between the SPCM (+) and SPCM (−) creams at 24 h after treatment. Therefore, the antipruritic effect of SPCM (+) cream was not dependent on the anti‐inflammatory effect of steroids but was largely attributed to characteristic features of the physical properties of SPCM, including its lamellar structure. Epidermal barrier dysfunction is observed in the skin of patients with AD in lesion and non‐lesion areas [45]. Therefore, proactive therapy using moisturisers and low‐dose topical anti‐inflammatory drugs is important for maintaining a healthy state of the skin barrier in the early stages before skin lesions become apparent. Therefore, PVA‐containing creams that provide alternative SC by SPCM may be promising tools for proactive therapy.

Protease activity in the skin of AD mice was significantly reduced in the SPCM (+) cream‐treated group. PAR2 is activated by proteolytic cleavage of its extracellular N‐terminus and is associated with eczema‐like inflammation and pruritus [46]. Epidermal protease activity is derived from endogenous or exogenous sources. Kallikrein 5 (KLK5) is a major endogenous protease that is involved in SC formation. KLK5 is upregulated in the SC of patients with AD, and its overexpression results in increased scratching via PAR2 activation [47, 48]. However, in the present study, mRNA expression of *KLK5* in skin was not altered by the application of SPCM (+) cream (Yokota et al. unpublished observations). The main allergens from house dust mites possess serine or cysteine protease activity [5]. These exogenous proteases penetrate the skin by degrading the epidermal barrier and inducing itching via PAR2 stimulation [34]. These results imply that the SPCM (+) cream protects the epidermis from protease allergen invasion, thereby inhibiting skin protease activity. This idea was further supported by the fact that the level of TSLP that is transcribed downstream of PAR2 signalling was relatively low in the SPCM (+) cream‐treated group.

In conclusion, the lamellar structure‐forming SPCM (+) cream may act as a substitute for the SC as it enhances skin barrier function and anti‐inflammatory and antipruritic effects. The SPCM (+) cream has promising therapeutic potential to prevent barrier‐disrupted skin diseases such as AD and xerosis.

## Author Contributions

Conceptualization: Masafumi Yokota, Tomohiro Matsumoto, Akane Kawamoto, Kumiko Dojo. Methodology: Masafumi Yokota, Tomohiro Matsumoto, Akane Kawamoto, Kumiko Dojo. Validation: Masafumi Yokota, Tomohiro Matsumoto, Akane Kawamoto, Kumiko Dojo, Sumika Toyama, Catharina Sagita Moniaga. Formal analysis: Masafumi Yokota, Tomohiro Matsumoto, Akane Kawamoto. Investigation: Masafumi Yokota, Tomohiro Matsumoto, Akane Kawamoto, Kumiko Dojo, Sumika Toyama, Catharina Sagita Moniaga. Resources: Noriyasu Ota, Mitsutoshi Tominaga, Kenji Takamori. Data curation: Masafumi Yokota. Writing – original draft: Masafumi Yokota; Writing – review and editing: Masafumi Yokota, Kenji Takamori. Visualisation: Masafumi Yokota, Tomohiro Matsumoto, Akane Kawamoto. Supervision: Junko Ishikawa, Daiki Murase. Project administration: Noriyasu Ota, Mitsutoshi Tominaga, Kenji Takamori. Funding acquisition: Noriyasu Ota. All authors read and approved the final manuscript.

## Conflicts of Interest

MT and KT received joint research funds from Kao Corporation. The other authors declare no conflicts of interest.

## Supporting information


Data S1.

## Data Availability

RNA‐seq data have been deposited in the DNA Data Bank of Japan (DDBJ) Sequenced Read Archive (https://www.ddbj.nig.ac.jp/) as follows: BioProject: PRJDB16927, Run: DRR512897‐DRR512905. Other data related to this article are available from the corresponding author on reasonable request.
