# Supplementary material for: Skin‐Protective Performance of Alternative Stratum Corneum Formed by a Pseudo‐Ceramide‐Containing Steroid Lamellar Cream
Source: Exp Dermatol. 2025 Mar 11;34(3):e70041. doi: 10.1111/exd.70041 (PMC11894917; doi:10.1111/exd.70041)

**Supporting Information**

**Skin-protective performance of alternative stratum corneum formed by a pseudo-ceramide-containing steroid lamellar cream**

Masafumi Yokota^1, 3^, Tomohiro Matsumoto^4^, Akane Kawamoto^3^, Kumiko Dojo^4^, Sumika Toyama^1^, Catharina Sagita Moniaga^1^, Junko Ishikawa^4^, Daiki Murase^3^, Noriyasu Ota^4^, Mitsutoshi Tominaga^1^, Kenji Takamori^1, 2^

^1^Juntendo Itch Research Center (JIRC), Institute for Environmental and Gender-Specific Medicine, Juntendo University Graduate School of Medicine, Chiba, Japan

^2^Department of Dermatology, Juntendo University Urayasu Hospital, Chiba, Japan

^3^Biological Science Research, Kao Corporation, Kanagawa, Japan

^4^Biological Science Research, Kao Corporation, Tochigi, Japan

*Corresponding author*

Kenji Takamori: Juntendo Itch Research Center, Institute for Environment and Gender-Specific Medicine, Juntendo University Graduate School of Medicine, 2-1-1 Tomioka, Urayasu-shi, Chiba 279-0021, Japan

TEL: + 81-47-353-3171

FAX: + 81-47-353-3178

E mail address: [ktakamor@juntendo.ac.jp](mailto:ktakamor@juntendo.ac.jp)


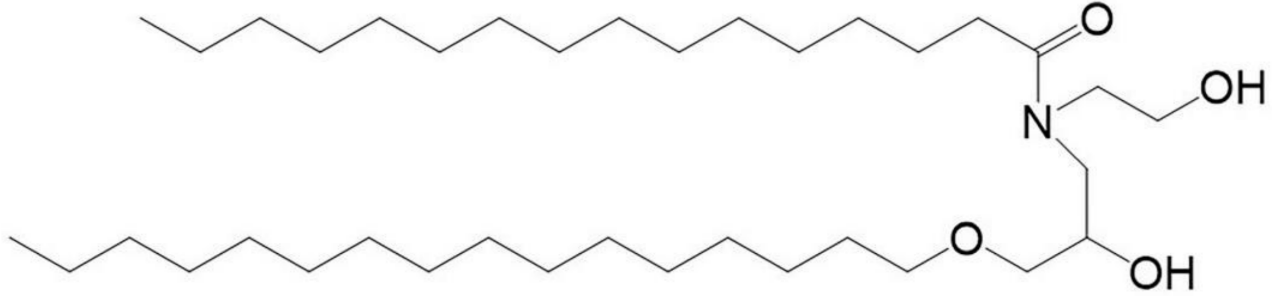


**Figure S1. Structure of synthetic pseudo-ceramide for medical (SPCM)**

IUPAC name, *N*-(3-hexadecoxy-2-hydroxypropyl)-*N*-(2-hydroxyethyl)hexadecanamide; molecular weight, 598 (C_37_H_75_O_4_N).


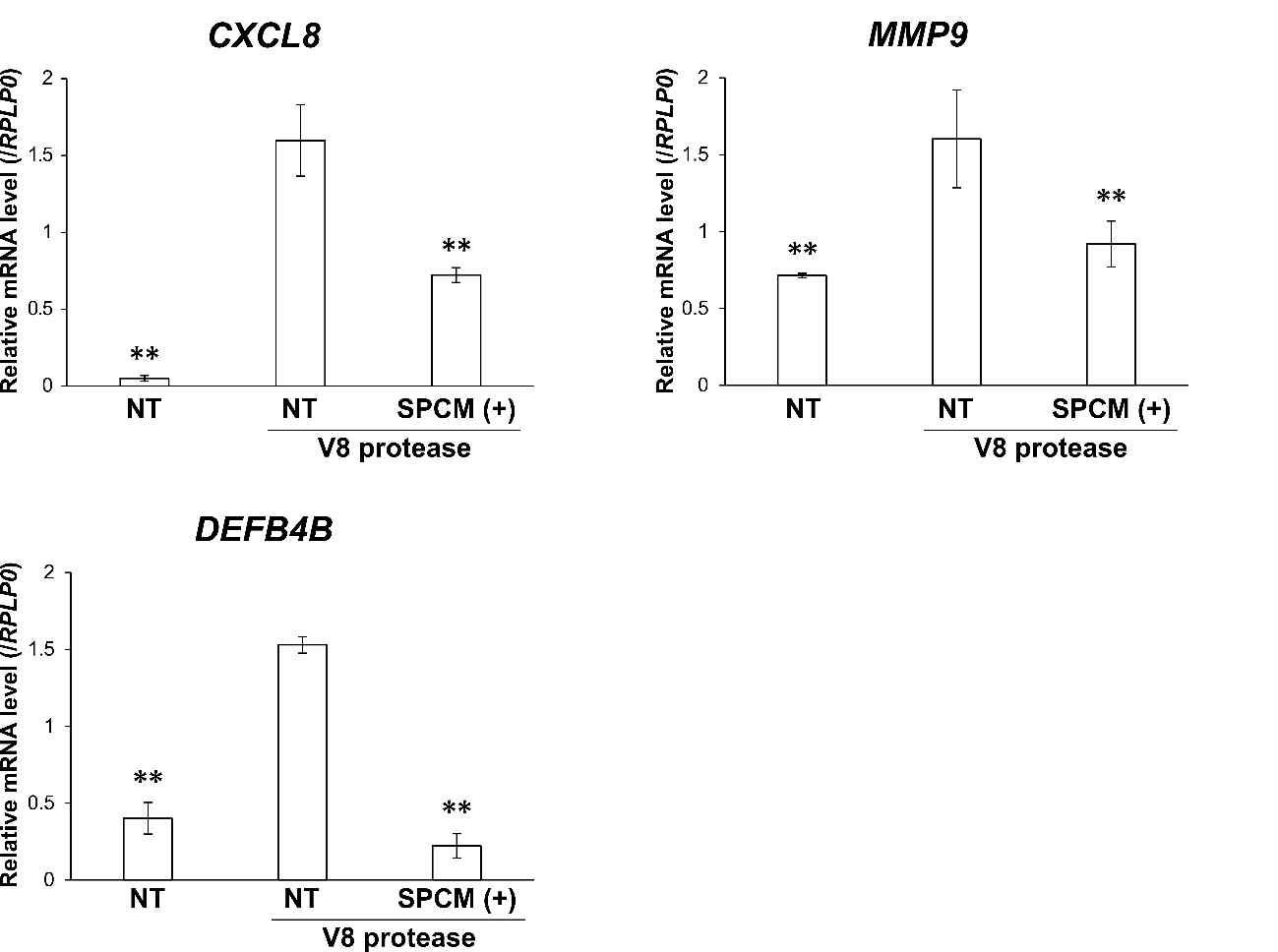


**Figure S2. mRNA expression changes of *CXCL8*, *MMP,* and *DEFB4B* by V8 protease stimulation**

mRNA expression was validated by quantitative real-time PCR (n = 3). Data represent mean ± SEM. ***P* < 0.01, Dunnett’s test (vs. NT under V8 protease challenge). CXCL8, C-X-C motif chemokine ligand 8; DEFB4B, defensin beta 4B; MMP9, matrix metalloproteinase 9; NT, untreated sample; SPCM (+); SPCM-containing PVA cream; SPCM, synthetic pseudo-ceramide for medical.

**
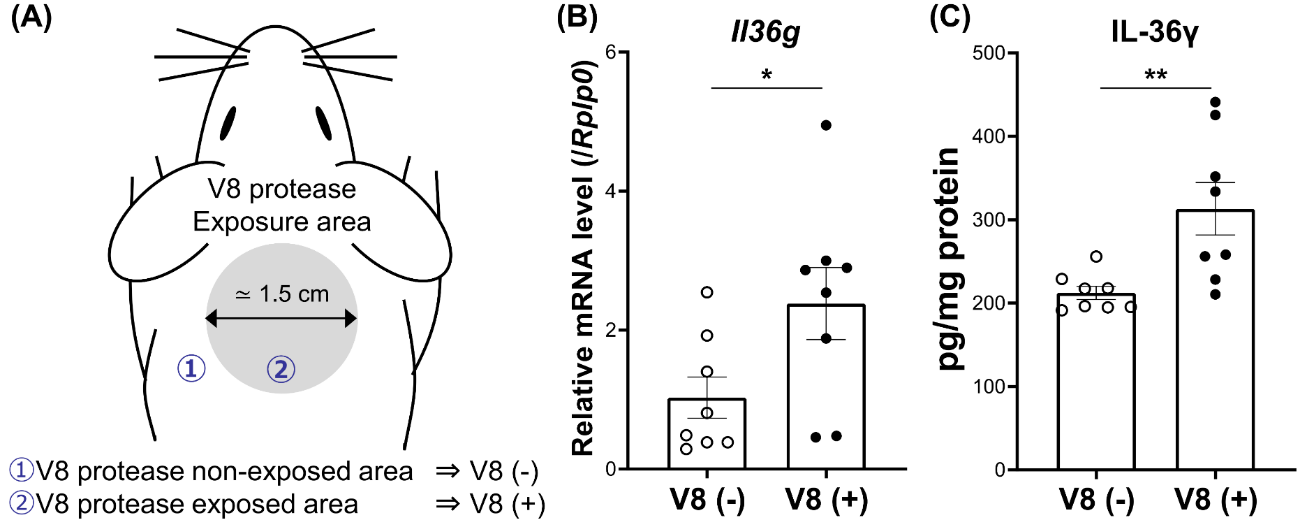
**

**Figure S3. IL-36γ expression in the mouse skin stimulated by V8 protease challenge**

(A) Scheme of the experiment. (B, C) *Il36γ* mRNA (B) and IL-36γ (C) expression at the V8 protease-exposed [V8 (+)] and untreated area [V8 (-)] in the same mouse (n = 8). Data represent mean ± SEM. **P* < 0.05, ***P* < 0.01, Student’s *t*-test. IL-36γ, interleukin-36γ.

**Table S1. Measurement and analysis parameters in MicroAct for detecting scratch motions**


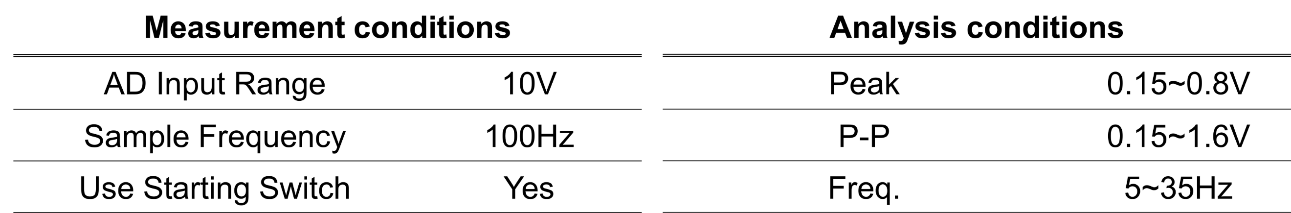

Supplement: Supplementary file 1 — Data S1. [file EXD-34-e70041-s001.docx]
